# Uniaxial Tensile Creep Behavior of Epoxy-Based Polymer Using Molecular Simulation

**DOI:** 10.3390/polym13020261

**Published:** 2021-01-14

**Authors:** Xueliang Li, Xiaoyu Zhang, Jianzhong Chen, Li Huang, Yong Lv

**Affiliations:** 1 School of Science, Wuhan University of Technology, Wuhan 430070, China; xueliang@whut.edu.cn (X.L.); cjzwhut@163.com (J.C.); wgdHL@163.com (L.H.); lvyonghl@163.com (Y.L.); 2 Hubei Key Laboratory of Theory and Application of Advanced Materials Mechanics, Wuhan University of Technology, Wuhan 430070, China

**Keywords:** creep behavior, epoxy polymer, service temperature, stress level, molecular dynamics

## Abstract

Based on the all-atomic molecular dynamics simulation method, the tensile creep behavior of epoxy-based polymer was discussed. The physical and mechanical properties of the model were characterized, such as glass transition temperature and yield strength. The simulation results are very close to the previous simulation and experimental results, and the correctness of the model is verified. On this basis, the tensile creep behavior and free volume evolution of polymer epoxy resin at different temperatures and stress levels were studied. The model fully predicted the three classical stages of epoxy resin creep (the primary, secondary and tertiary) and the dependent behavior of epoxy resin creep on temperature and stress level at the molecular level, and the creep rate increases with the increase of temperature and stress level. It was found that with the progress of the creep process, the proportion of free volume increases gradually under high stress levels, indicating that the effect of creep behavior on the structure of epoxy resin is that the interaction between atoms becomes weaker and weaker by increasing the distance between atoms, which finally induces creep failure in the material.

## 1. Introduction

Fiber-reinforced composites based on epoxy resin have been widely used in industrial production because of their high specific strength, high specific modulus, strong design ability, good overall formability and so on [1]. As a major structural material, its stability has increasingly become the focus of attention. However, as we all know, its mechanical behavior depends on time, and it will show creep behavior with the passage of time, which is mainly due to the creep of viscoelastic epoxy resin matrix. Thus, understanding the mechanical behaviors and properties of such materials under various physical conditions is of crucial importance. Epoxy resin polymers with high cross-linking and excellent mechanical properties are prepared by cross-linking reaction of epoxy resin and curing agent. A noteworthy point is the creep of epoxy-based polymers with high cross-linking is also greatly affected by temperature, stress level, strain rate, humidity and other environmental factors. Therefore, it is particularly important to study the creep properties of cross-linked epoxy resin in detail and systematically.

Theoretical and experimental investigations on creep behaviors of epoxy matrix composites were extensive at the macroscopic length scale. J. Raghavan et al. [2] presented a model incorporating a modified thermal activation theory to model and predict creep of polymer composites, which predicted the creep of a unidirectional, continuous carbon fiber reinforced polymer composite and its epoxy matrix, over a wide range of stress (10–80% of ultimate tensile strength) and temperature (295–433 K). They concluded that the reinforcing carbon fibers do not alter the creep mechanism but do alter the creep behavior of the epoxy matrix, resulting in reductions in creep rate and in the magnitude of creep from the analysis of model parameters. Yi-Luen Li et al. [3] performed the experiments to test the creep behaviors of carbon fiber (CF)/epoxy resin thermosetting composites and MWCNTs (multi-walled carbon nanotubes) /CF/epoxy resin composites at different stresses, orientations of fiber, temperatures and humidities and found that creep strain decreased as the number of cycles in cyclic creep tests increased at room temperature and at a high temperature of 55 °C. Inés Costa et al. [4] revealed that this model is capable of predicting with very good accuracy the long term behavior of concrete structures with carbon fiber reinforced polymer (CFRP) systems up to a sustained stress level of 60% of the adhesive’s tensile strength by fitting to the experimental creep curves with the modified Burgers model. Nicolas Candau et al. [5] investigated the effect of the strain rate on damage in carbon black filled ethylene propylene diene monomer rubber (EPDM) stretched during single and cyclic uniaxial loading and conducted that increasing the strain rate in cyclic loading conditions yields in a filler network accommodation and a high self-heating whose combined effect is proposed as a possible cause of the ability of filled EPDM to limit damage by reducing cavities opening during loading, and favoring cavities closing upon unloading. Yunlong Jia et al. [6] investigated the creep response of a unidirectional flax fiber reinforced biobased epoxy under various tensile stress levels and showed that unidirectional flax fiber composites exhibit perceivable creep at low stress levels and have a relatively short creep rupture life at high stress levels. Besides, creep at high stress are characterized with a development of extensive damage events and doesn’t show typical three-stage creep deformation behavior over creep life. The experiments carried out in the studies of creep behaviors mentioned above are of the macroscopic scale, while principles and mechanisms of creep are to be explored at the molecular scale, which is an extremely small scale that often hinders experiments. At the same time, most of the previous studies on the creep performance of epoxy resin matrix are based on the tensile creep of epoxy-based composites, and there are few studies on the creep mechanism and mechanical properties under different service environments of epoxy resin itself.

With the maturity of computer simulation technology, it provides useful supplementary research methods for the study of polymer creep properties. Molecular dynamics is a general method for simulating the behavior of atoms by iteratively solving Newtonian equations. Now it has been established and has been successfully applied to material simulation in many fields [7,8,9]. A series of pilot works have been done by researchers before in various aspects including model construction, physical properties characterization and mechanical testing on cross-linked epoxy resins [10,11,12,13,14]. A.L. Bowman et al. [15] investigated the structural and free volume evolution (correlated with damage) during creep of model amorphous polyethylene at various applied stress states (tension, shear, compression), stress levels and temperatures, showing that the phenomenological macroscale creep response of polyethylene (PE) can be captured through molecular dynamics (MD) simulations. They conducted that the transition from glassy to rubbery state is reflected in the chain dynamics and damage evolution by simulations performed at temperatures below and above *T*_g_. By finding the correlation between the steady-state creep rate and steady-state void nucleation rate, they considered that secondary creep is heavily driven by void nucleation, while tertiary creep is driven by void growth and coalescence. Hyungbum Park et al. [16] conducted the cyclic loading–unloading simulations at two different frequencies using atomistic models for epoxies cured by aliphatic and aromatic curing agents, triethylenetetramine (TETA) and diethyltoluenediamine (DETDA), respectively. They studied the structure and found that irreversible dihedral angle transitions near the benzene ring of the curing agent DETDA were responsible for low ratcheting resistance and stiffness degradation during the cyclic creep deformations. Penghui Cao et al. [17] simulated the molecular processes of creep in metallic glass thin films at experimental timescales using a metadynamics-based atomistic method and validated the relevance of two original models of the mechanisms of amorphous plasticity: one focusing on atomic diffusion via free volume and the other focusing on stress-induced shear deformation. The above research proves the applicability of MD simulation in studying the molecular model, mechanical behavior and creep evolution of polymers. However, the mechanical properties and related processes of the cross-linked epoxy resin during the creep process are still unclear, especially the creep behavior under a complex and extreme service environment.

Taking into account the information previously exposed, and in order to address the lack of creep research in this topic of epoxy-based polymer, the tensile creep behaviors of cross-linked epoxy polymers under constant uniaxial tensile stress were investigated by MD simulations considering the influence of different service temperatures and stress levels. Based on the theory of molecular dynamics, the all-atomic model of EPON862/TETA (diglycidyl ether of bisphenol F/triethylenetetramine) epoxy resin polymer was established. In this paper, the details and process of the simulation and the related calculation methods are introduced in detail. Based on the established molecular dynamics model, the physical and mechanical properties such as glass transition temperature, yield strength and Young’s modulus of epoxy resin polymers were calculated, and the creep behavior of epoxy resin at different service temperatures and stress levels was simulated. The curve of true strain with time during creep was drawn, and the variation of creep rate with temperature and stress was studied. The simulation results reveal the temperature-and stress-dependent behavior of epoxy resin creep at the molecular level, and explore the evolution of free volume during creep. The study of the creep behavior of EPON862/TETA epoxy resin polymers is helpful for us to understand this behavior more comprehensively from a microscopic point of view and to further explore the creep mechanism of epoxy resin.

## 2. Simulation Details

### 2.1. General Method of Model Construction

The commercial software package Materials Studio version 17.0 (MS 17.0) produced by the American Accelrys Company and the parallel molecular dynamics code large atomic molecular massively parallel simulation (LAMMPS) distributed by Sandia National Laboratories were used to carry out epoxy resin molecular modeling and tensile creep simulation respectively. During MD simulation, the molecular interaction is simulated by employing functional forms and force field parameters defined in consistent valence force field (CVFF) [18]. The applicability of CVFF potential in the investigation of epoxy-bonded system has been validated in previous simulation studies [19,20]. The modeling process are presented subsequently.

The curing agents triethylenetetramine (TETA) and the diglycidyl ether of bisphenol F (EPON862) were used to build the structure of epoxy polymer. First, the molecular monomers of EPON862 and TETA were established and their structures were optimized respectively. The specific optimization process is as follows. The force field of COMPASS was adopted to optimize the structures by using the energy optimization and geometric optimization function of Forcite Calculation module in MS 17.0. In the molecular modeling, the molecular polymerization degree of all epoxy resin monomers was set to 0. In fact, as a simplified model, *n* = 0 is a reasonable approximation.

**Figure 1:**
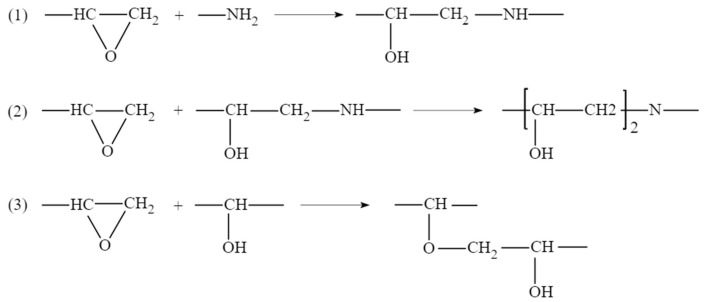
Three principal reactions involved in the curing of a diamine with a diepoxide compound.

**Figure 2:**
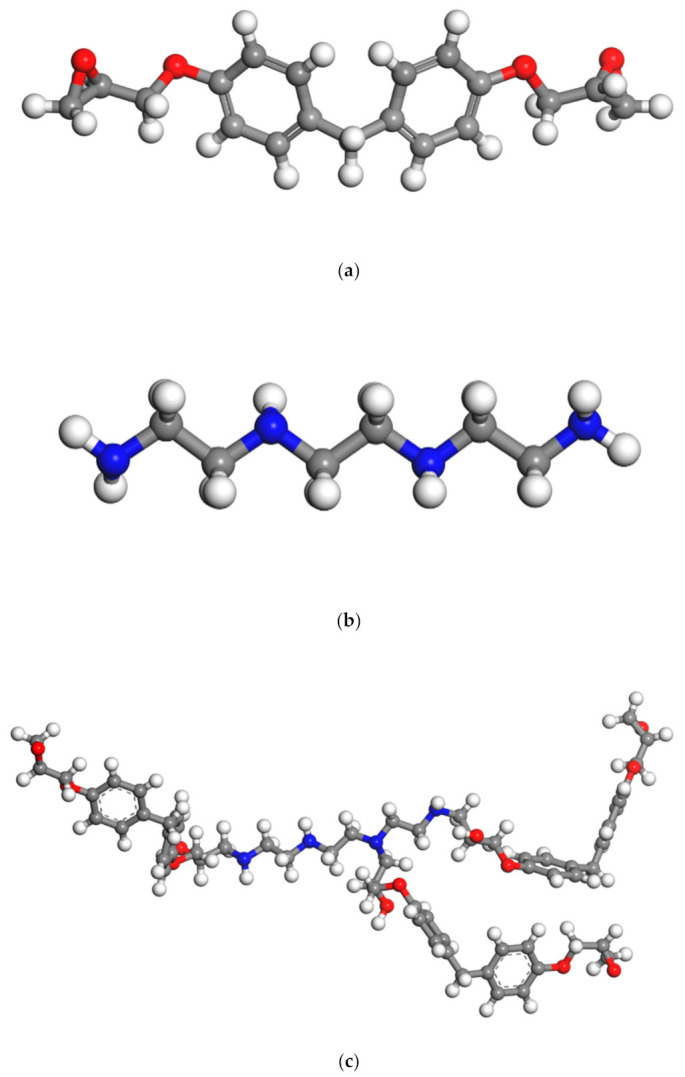
Illustration of (**a**) molecular structure of the considered epoxy resin (EPON862) and (**b**) curing agents (TETA) and (**c**) prepared representative monomer of EPON862/TETA (diglycidyl ether of bisphenol F/triethylenetetramine). Oxygen: red; carbon: gray; nitrogen: blue; hydrogen: white.

**Figure 3:**
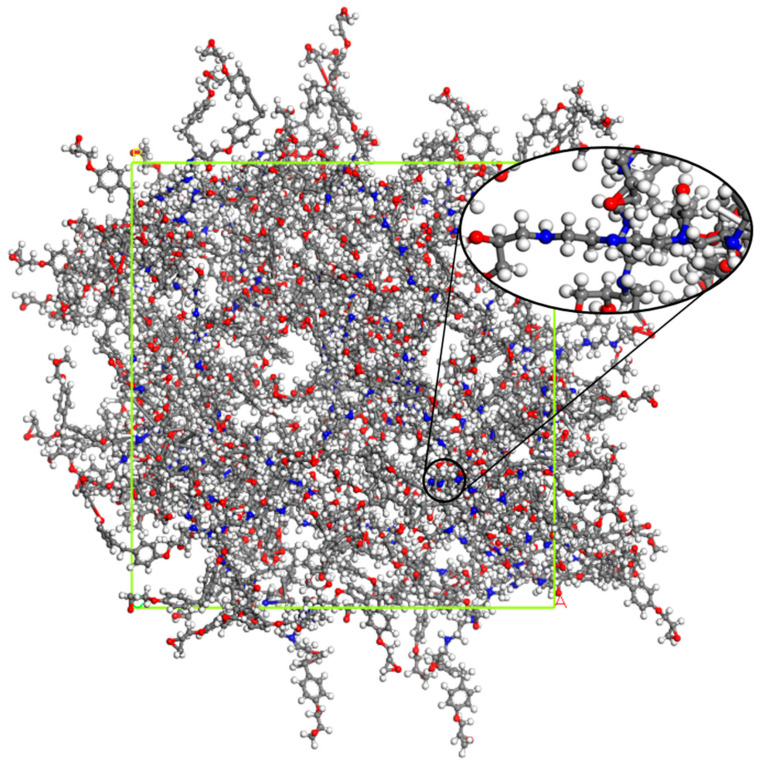
Cross-linked model of EPON862/TETA cell with 60 representative monomers (9420 atoms in total), all atoms are in stick and ball representation. Oxygen: red; carbon: gray; nitrogen: blue; hydrogen: white.

After the above process of monomer construction, the cross-linking reaction of the structure can be performed with a method of representative molecule. The crosslinking reaction mechanism of epoxy curing agent has been studied a lot before [21,22,23]. Generally acceptable is the mechanism shown in the Figure 1, which includes three main reactions: ring-opening addition reaction of epoxy group with ring-opening primary amine to form secondary amine; secondary amine reacts with epoxy group to form tertiary amine; at high temperature or in the presence of catalyst, the hydroxyl groups on the secondary amine and tertiary amine adduct can react with epoxy group [14]. The TETA used in this paper is an amine curing agent containing only primary and tertiary amines, so the first two processes were considered only. Therefore, a method of representative molecule was used to make EPON862 and TETA curing agent cross-linked into a long chain called EPON862/TETA representative monomer and geometry optimized by the same method as above. The chemical structures of these two monomers and the EPON862/TETA representative monomer are presented in Figure 2. Next, a three-dimensional periodic unit cell consisting of 60 EPON862/TETA representative monomers with an initial density of 0.8 g/m^3^ was constructed by using the MS Amorphous Cell module with COMPASS force field temporarily. Resulting atomistic unit cells comprised a total of 9420 atoms for EPON862/TETA structure. As a result, the modeled epoxy structure has the size of 5.13 nm × 5.13 nm × 5.13 nm. The chemical structure of EPON862/TETA and local enlarged drawings are shown in Figure 3. So far, the initial molecular model of epoxy resin polymer had been constructed. In this paper, during the process of molecular simulation, Andersen and Berendsen methods were used to control the temperature and pressure respectively, and atom-based and Eward methods were used to calculate van der Waals and electrostatic interactions, respectively. After the initial construction and optimization process, the model was minimized within LAMMPS using the CVFF potential and then equilibrated with a 1 fs time steps using NPT dynamics at 300 K and zero pressure for about 200 ps until the temperature, pressure, density, and total energy of the system fluctuated around a constant value. The final simulated density of the EPON862/TETA was 1.134 g/m^3^, exhibiting a good agreement with previous studies compared with previous MD and experimental studies in Table 1. 

Based on the constructed epoxy models, this paper used uniaxial stretching to study the creep behavior of epoxy resin. In the specific simulation process, different levels of constant stress levels lower than the yield strength were applied along the X direction of the epoxy resin, while ensuring that the stress levels in the other two directions were zero to simulate the creep process of uniaxial tension. A study of temperature dependence was carried out by selecting a series of temperature ranges under the same constant stress level to simulate the creep process of epoxy resin with temperature changes.

### 2.2. Physical and Mechanical Properties

**Figure 4:**
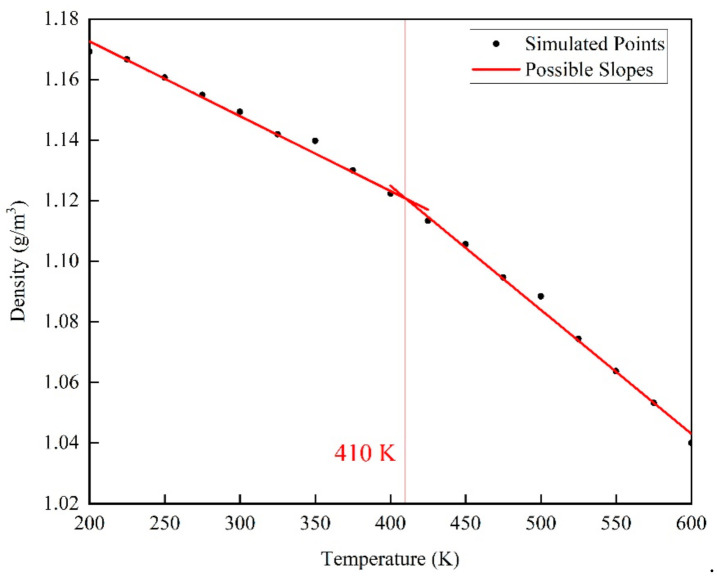
Density versus temperature relationship for cross-linked EPON862/TETA model: the intersection of fitted lines for the low and high temperature range indicates the glass transition temperature.

After the EPON862/TETA model construction, its glass transition temperature (*T*_g_) was calculated, where the density was recorded at varied temperature conditions applied in NPT equilibration. Specifically, the equilibration process was carried out at a temperature rising from 300 K to 500 K at a rate of 1 K/ps for 200 ps, and lasting for 500 ps at 500 K. After that, the equilibration process was performed with the temperature decreasing to 200 K at a rate of 1 K/ps for 300 ps. Then, the equilibration process was performed with the temperature rising from 200 K to 600 K. The equilibration was run for 500 ps at each temperature increase of 25 K, and the density was recorded every 1 ps, and the last 50 ps of density data of were averaged to calculate density of the epoxy model in a steady level during the process. The relationship between density and temperature is shown in Figure 4. Glass transition temperature was calculated by finding the intersection point between two fitting lines against the density-temperature plot.

After the glass transition temperature of the model was simulated, the Young’s modulus and yield strength of the epoxy resin model were determined. To better understand the creep behavior of epoxy, the uniaxial tensile simulation s was conducted by stress-controlled deformation. Young’s modulus was obtained from the uniaxial tensile deformation and could be calculated based on the relatively linear part of the stress–strain curve, as shown in Equation (1).

(1) $$E=\frac{\sigma_{XX}}{\varepsilon_{XX}}$$

**Figure 5:**
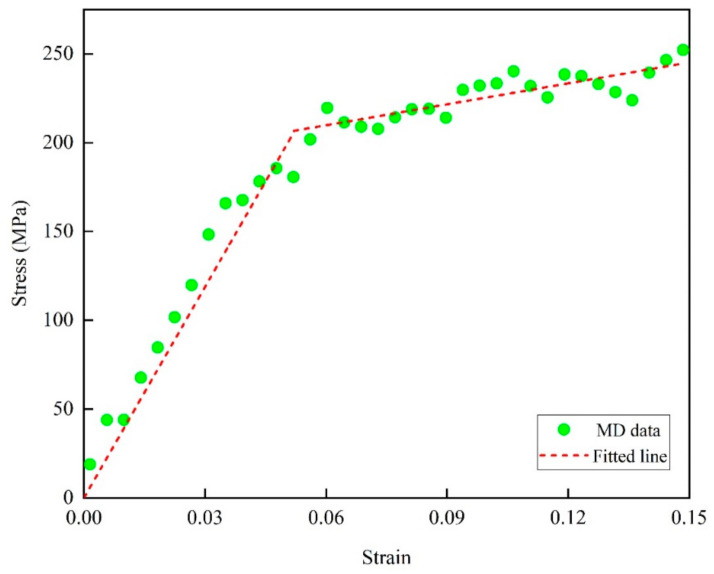
Simulated uniaxial tensile stress–strain profiles for EPON862/TETA epoxy system.

As a large strain rate can influence stress response in uniaxial tensile deformations, a small strain rate was used to approach the quasistatic mechanical test [10]. Therefore, in order to determine the stress levels applied on the epoxy resin, uniaxial tensile deformation in the X direction up to 0.15 strain was carried out at a strain rate of 3 × 10^8^/s (see Figure 5) at 300 K in an NPT ensemble for 500 ps, while the Y and Z directions were zero pressure. The yield strength of the polymer could be obtained by fitting the flow stress curve obtained by uniaxial tensile test. For the strain behavior of epoxy resin polymers under small deformation (plastic strain less than 0.2), many scholars have proposed various extrapolation models, such as the Hollomon model, Swift model, Ludwik hardening model, etc. The Ludwik hardening model has been used to successfully simulate the corresponding mechanical properties of the polymer by other authors [15]. Ludwik’s hardening model derived from Hollomon’s model is a typical unsaturated extrapolation model of fixed initial values, which ensures that the plastic strain segment passes through the yield point of the material. Based on the Ludwik’s hardening model and previous research work, the yield stress of the epoxy resin structure was determined by fitting the linear elasticity and Ludwik’s hardening model, and the expression is as follows:

(2) $$\sigma =\sigma_{y}+h{\left(\varepsilon_{p}\right)}^{n}$$

where *σ_y_*, *h*, *ε_p_* and *n* are the yield stress, strength coefficient, plastic strain, and hardening exponent respectively. The yield point was chosen as the point at which the deviation between the raw MD data and elastoplastic model was minimized. 

## 3. Results and Discussion

### 3.1. Basic Characteristics of the Epoxy Model

The glass transition temperature represents the temperature at which a polymer changes from a highly elastic state to a glass state and refers to the transformation of an amorphous polymer (including the noncrystalline part in a crystalline polymer) from a glass state to a highly elastic state or from the latter to the former. The glass transition temperature is the lowest temperature at which the macromolecular segment of the amorphous polymer moves freely. To determine the appropriate temperature ranges for the creep simulations, the glass transition temperature was first calculated from the change of slope of the density versus temperature response as shown in Figure 4. The average density values in the temperature range of 200 K to 400 K and 425 K to 600 K were fitted linearly, and two fitting lines of different slopes with fitting degrees of 0.985 and 0.996 were obtained.

From Figure 4, a series of points were obtained after averaging, and finally we get the glass transition temperature of the epoxy resin structure about 410 K from the curve obtained after corresponding fitting. This glass transition temperature is basically consistent with the results of other researchers through molecular dynamics simulations [19]. This result shows the rationality of the epoxy resin model structure established in this article, and the appropriate temperature can be selected for further research based on this. In this paper, six different temperatures were selected under and above *T*_g_ (410 K), 298 K, 323 K, 363 K, 393 K, 423 K, 453 K, and the creep behavior of epoxy resin was simulated more systematically by uniaxial constant stress tension in the temperature range of 298–453 K. 

From Figure 5, the yield strength of the resulting epoxy resin model is 206.64 MPA, and the corresponding strain is 0.052. It should be pointed out that the yield strength 206.64 MPa of epoxy resin simulated here is slightly lower than the 264.26 MPa reported in previous studies, which may be due to the fact that the cross-linking degree of the model established using the method of representative monomer in this paper is slightly lower than that of the previously reported model (81.3%) [16]. At the same time, through postprocessing to fit the points obtained in the linear stage of the stress-strain curve in the tensile process, we found that the Young’s modulus of the epoxy resin system was 4.390 ± 0.251 GPa, with the final fitting degree R^2^ equaling 0.98. Based on the macroscopic tensile creep experiment and polymer simulation results of previous researchers, it is important to select a set of appropriate and representative stress levels to analyze the creep behavior of epoxy resin polymers. Before the final stress level was determined, a wide range of stress levels of 5–95% yield strength was simulated. According to the simulation results, we found that the strain value generated at the same time and temperature under the excessively low stress level was too small to observe a more obvious creep curve. At the same time, it lasted for quite a long time in the second stage of tensile creep with an excessively low stress level. In order to analyze the effects of different stress levels on the properties and molecular structure of epoxy resin during tensile creep, the stress levels applied in this paper were set at 25%, 50%, 60%, 70%, 85% and 95% of the yield strength. 

### 3.2. Temperature Dependence

**Figure 6:**
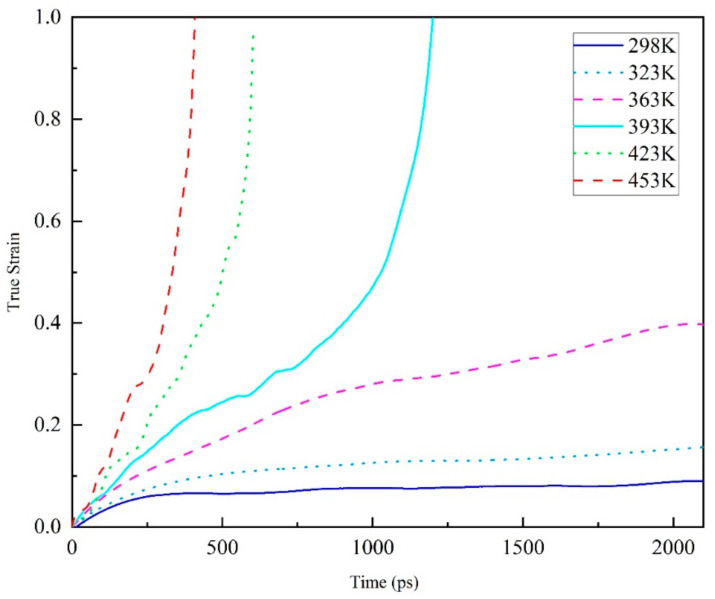
The true strain versus time of creep behavior during tension at 100 MPa (about 50% of yield strength) of an amorphous polyethylene system (60 EPON862/TETA monomers, 9420 atoms total) under various service temperatures (298–453 K).

In Figure 6, the overall tensile creep behavior at 100 MPa of the amorphous EPON862/TETA system (see Section 2.1 for a description of the model) under various service temperatures is shown. As is shown, the true strain vs time is given.

Although the time scale of simulating creep response in this paper was many orders of magnitude smaller than that of the macroscopic creep experiment, the creep curve of molecular simulation shows the same trend as the macroscopic creep of polymer, exhibiting primary, secondary, and tertiary creep stages. Besides, the creep rate increases with the increase of temperature. As seen Figure 6, when the service temperature is lower than the glass transition temperature, the creep curve of the epoxy resin polymer model shows the classical macroscopic creep experimental curve, but the variation curve of the real strain with time only shows the first stage. However, when the service temperature is higher than the glass transition temperature, the tensile creep curve shows a complete creep stage, that is, the first stage, the steady-state creep stage and the third stage. At the same time, after exceeding the glass transition temperature, with the increase of temperature, the real strain changes faster and faster with time at 423 K and 453 K, which is quite different from that at 423 K. This shows that near the glass transition temperature, the transformation of epoxy resin polymer from glass state to rubber state accelerates the creep process, which leads to the decrease of material life. Specifically, glass transition is a transition between a high elastic state and glass state, indicated by the molecular structure. The glass transition temperature is a high-polymer amorphous part of the frozen state to thaw a slack phenomenon, and not like a phase transition phase change heat, so it is not a primary phase change nor secondary phase transition (polymer dynamic mechanics according to the main shift). Under the glass transition temperature, the polymer is in the glass state, the molecular chain and the chain segment cannot move, but the atoms (or groups) which constitute the molecule vibrate in its equilibrium position. At the glass transition temperature, although the molecular chain cannot move, the chain segment starts to move, showing high elastic properties. If the temperature rises again, the entire molecular chain will move and show viscous flow properties. Based on the above discussion, this simulation results reveals the temperature and time-related behavior of epoxy resin polymer creep at the molecular level.

### 3.3. Stress State Dependence

**Figure 7:**
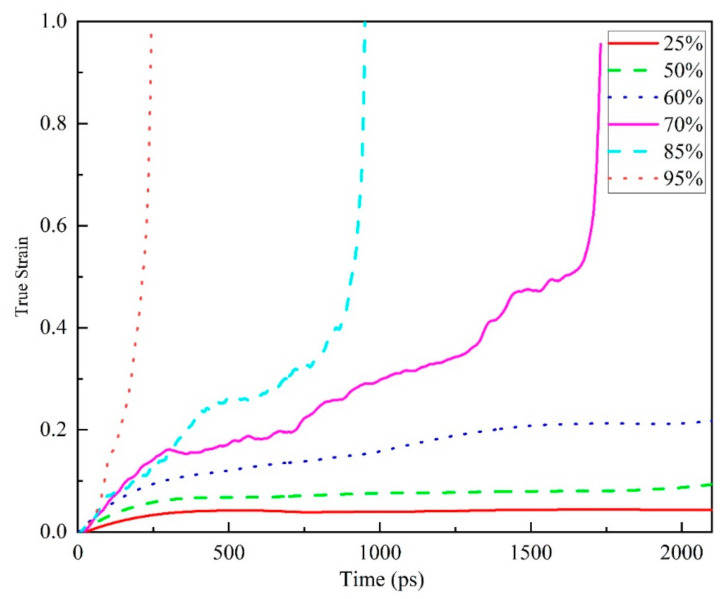
The true strain versus time of creep behavior during tension at 298 K (about 0.73 *T*_g_) of EPON862/TETA system (60 EPON862/TETA monomers, 9420 atoms total) under various applied stress levels (50–200 MPa, 25–95% of yield strength).

Figure 7 shows the overall tensile creep behavior of amorphous polymer epoxy resin at different stress levels at 298 K. The creep time curve shows the common trend of epoxy resin creep, such as the creep curves of three different stages similar to the previous temperature-dependent study, and with the increase of stress level, the faster the strain of epoxy resin changes under high stress. By analyzing the curve of creep with time, the relationship between the stress level and the creep of epoxy resin can be ascertained. In a certain simulation time, at a lower stress level (less than or equal to 60% yield strength), the creep curve only shows the initial and second stages; at higher stress levels, three complete creep stages can be seen clearly. The time required for the third stage to appear becomes shorter with the increase of the stress level, compared with the traditional macroscopic creep experiments whose period is longer and the third stage is more difficult to be obtained. This result provides us with the possibility of using molecular dynamics simulation to further study the creep mechanism of epoxy resin.

### 3.4. Creep Rate Upturn

**Figure 8:**
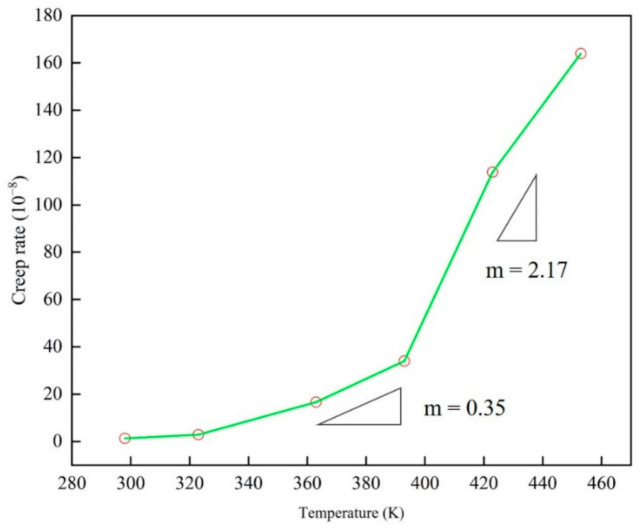
The variation curve of steady state strain rate versus temperature of epoxy resin model at different service temperatures (298–453 K).

**Figure 9:**
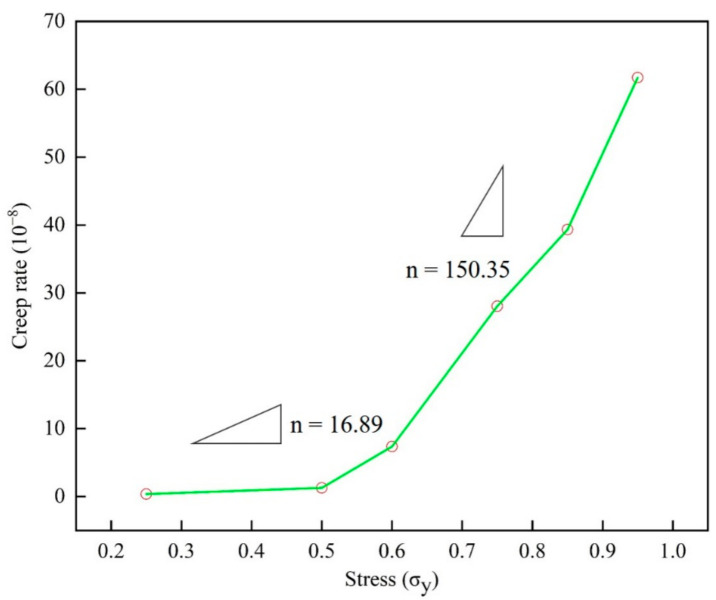
The steady-state strain rate versus temperature curve of epoxy resin model at different stress levels (25% to 95% of yield strength).

By simulating the creep curve of epoxy resin under different service temperature and stress by molecular dynamics, the steady-state creep rate at each temperature and stress was determined. In the calculation of steady-state creep rate in the second stage, origin software was used for linear fitting analysis of the linearity of each creep curve in this stage. The average linear fitting degree is up to 95% or more in the second stage, which ensures the correctness and reasonability of the results. Drawing the results shown in Figure 8 and Figure 9, the creep rate increases with the applied stress in a manner typical of polymers, and the nonlinear behavior of the creep rate was changing monotonously with different temperatures and stresses. 

In Figure 9, two peaks can be observed. The creep rate slope *n* is 16.89 at low stress, and in the high stress state, the value *n* increases to 150.35, where *n* is the stress index. Please note that the change of the exponent *n* here is consistent with the stress gap observed earlier. In Figure 8, the slope m of the creep rate is 0.35 below the glass transition temperature, and this peak increases to 2.17 when it is higher than the glass transition temperature. It should be pointed out that the change of the peak value here is consistent with the effect of the relationship between service temperature and glass transition temperature on the creep of epoxy resin. Here, the creep slope at different temperatures is similar to that under different stress states, indicating that there is also a temperature gap, which reveals the nonlinear relationship between creep rate and temperature.

In the experimental results of different materials, the transformation of creep rate behavior has been widely studied. It is generally believed that the thermal activation process under low stress is transformed into the process of stress activation due to the action of atomic diffusion and shear deformation. Through the discussion of Figure 6, Figure 7, Figure 8 and Figure 9, the creep rate behavior of epoxy resin with the coupling relationship between atomic diffusion and shear phase transformation can be discussed, so as to analyze the steady-state creep mechanism of epoxy resin.

### 3.5. Free Volume Evolution

The theory of free volume was first put forward by Fox and Flory, which holds that the volume of polymer materials consists of two parts: the volume occupied by molecules $V_{0}$ (that is the actual occupied volume) and the unoccupied volume $V_{f}$ (that is free volume). The latter is the gap between molecules, which is dispersed in the material in the form of “holes” and provides space for molecular activity, so that the molecular chain can move. 

**Figure 10:**
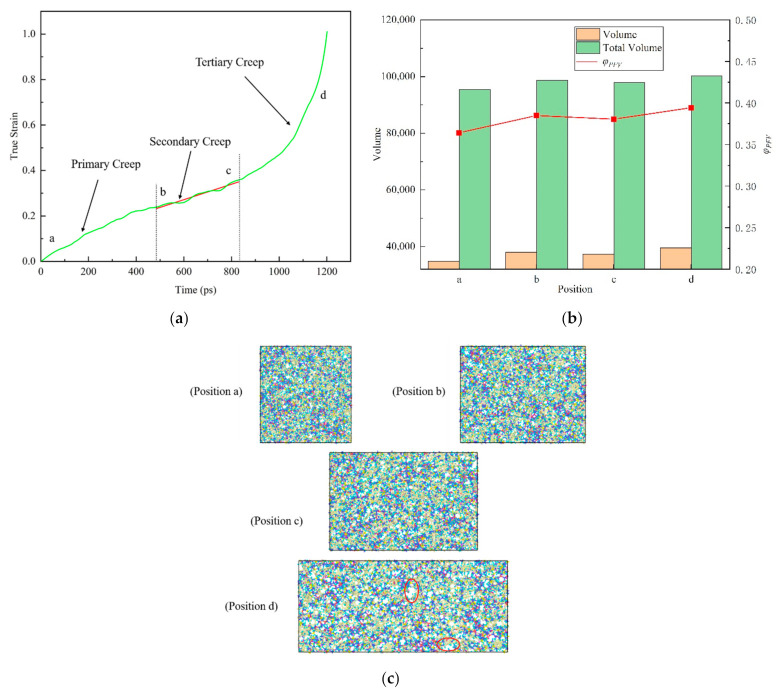
The relationship between real strain and time shows the different creep stages of amorphous polymer epoxy resin model near the glass transition temperature (at 393 K, about 0.96 *T*_g_) under 100 MPa tensile stress. (**a**) Three stages of creep (primary, secondary and tertiary). (**b**) Total volume, free volume and the proportion of free volume at the corresponding position of the model. (**c**) Snapshots of the atom at the corresponding location. The red ellipses represent the voids. Among them, a position represents the initial, b indicates the beginning of the second (steady-state) creep, c indicates the beginning of the third creep, and d indicates the rapid change of the real strain.

**Figure 11:**
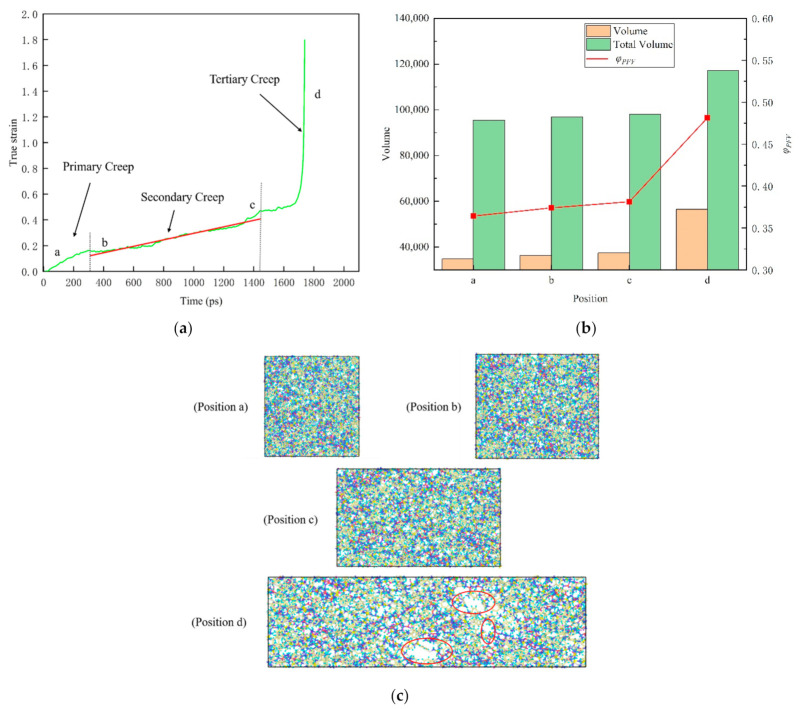
The relationship between real strain and time shows the different creep stages of amorphous polymer epoxy resin model in response to a high stress level (150 MPa, about 70% of yield strength) tensile stress at room temperature 298 K. (**a**) Three stages of creep (primary, secondary and tertiary). (**b**) Total volume, free volume and the proportion of free volume at the corresponding position of the model. (**c**) Snapshots of the atom at the corresponding location. The red ellipses represent the voids. Among them, a position represents the initial, b indicates the beginning of the second (steady-state) creep, c indicates the beginning of the third creep, and d indicates the rapid change of the real strain.

In order to give a further study of the physical mechanism of deformation of epoxy resin during tensile creep, we selected the change of free volume of epoxy resin during tensile creep at high temperature and high stress respectively. It should be pointed out that the free volume here was calculated by the quantum chemical wave function analysis program Multiwfn. Because the total volume and free volume of epoxy resin model constantly change during creep, the free volume of each system cannot be compared directly, the percentage of free volume ($\varphi_{PFV}$) is used to express the relative size of free volume, which is expressed as follows:

(3) $$\varphi_{PFV}=\frac{V_{f}}{V_{0}+V_{f}}$$

where $V_{0}$ is the actual occupied volume and *V_f_* is the free volume. In order to study the change of free volume in the creep process of epoxy resin model near the glass transition temperature under low stress and room temperature under high stress level, two typical creep processes were selected for analysis. As shown in Figure 10 and Figure 11, the time-varying curves of real strain of 393 K (near the glass transition temperature, about 0.96 *T*_g_) under constant tensile stress of 100 MPa and 298 K room temperature under high stress 150 MPa (about 70% of yield strength) are drawn respectively. The different creep stages of nonstatic polymer epoxy resin under high temperature and high stress were shown respectively, and three stages of creep (primary creep, secondary creep and tertiary creep) were observed.

In Figure 10b, the free volume ratio of epoxy resin does not show obvious monotonous change during creep near the glass transition temperature, showing a relatively stable state. This is due to the fact that the molecular chain of epoxy resin polymer is in the glassy state near the glass transition temperature, and the range of motion is so small that it is difficult to change the free volume. At the same time, we noticed that the free volume decreases during the change from b position to c position. This can be interpreted as preventing the flow of molecular segments of the material during deformation, when the material shows a brittle mechanical response. However, as is shown in Figure 11b, the evolution of free volume in tensile creep process under high stress at room temperature shows a different phenomenon. In detail, both the total volume and the free volume increase with the progress of the creep process. Further study found that the proportion of free volume $\;\varphi_{PFV}$ in the creep process of epoxy resin gradually increased. 

From Figure 10c and Figure 11c, the atomic snapshots at different positions in the creep process can further help us understand the change of free volume more intuitively. With the progress of tensile creep, the atomic spacing gradually increases to different sizes of voids. Based on the above, it can be inferred that with the gradual increase of the void, the epoxy resin polymer will have a softening effect and then lead to the failure process of material properties. The experiments reveal that the effect of creep behavior on the structure of epoxy resin is that the interaction between atoms becomes weaker and weaker by increasing the distance between atoms, which will lead to the final creep failure of the material.

## 4. Conclusions

In this paper, the EPON862/TETA epoxy resin polymer was modeled and simulated by MS and LAMMPS, and the physical and mechanical properties of the model were characterized, including density, glass transition temperature, yield strength and Young’s modulus. The simulated values of the above properties are mild and good with the simulation or experimental results of previous researchers. The simulation results reveal the dependence of the creep behavior of epoxy resin on temperature and stress level at the molecular level, and the creep rate increases with the increase of temperature and stress level. We observed that there is a temperature gap in the process of creep strain changing with time at different service temperatures. We found that, with the progress of the creep process, the proportion of free volume increases gradually under high stress level, indicating that the effect of creep behavior on the structure of epoxy resin is that the interaction between atoms becomes weaker and weaker by increasing the distance between atoms to finally induce creep failure in the material. The results of this study provide an important reference for studying the deformation mechanism and damage evolution of epoxy resin amorphous polymers during creep. Further work is required in exploring these simulated results presented here using a larger system. Moreover, it is highly desirable to compare these simulated results to the corresponding experimental data in the future.

## Figures and Tables

**Table 1:** Predicted density of the epoxy systems of epoxy resin polymer.

Property	Present Study	Previous Study	
		Experiment	MD
Density (g/cm^3^)	1.134	1.124 [24]	1.14 [25]

## Data Availability

The data presented in this study are available on request from the corresponding author.
